# Development of a short form of the Singapore Caregiver Quality of Life Scale – Dementia: SCQOLS-D-15

**DOI:** 10.1186/s41687-021-00330-0

**Published:** 2021-07-10

**Authors:** Chun Fan Lee, Dennis C. C. Seow, Irene Teo, Shirlyn H. S. Neo, Grace M. Yang, Geok Ling Lee, Wee Shiong Lim, Allyn Hum, Yin Bun Cheung

**Affiliations:** 1 Centre for Quantitative Medicine, Duke-NUS Medical School, Level 6, Academia, 20 College Road, Singapore, 169856 Singapore; 2 Department of Geriatric Medicine, Singapore General Hospital, Singapore, Singapore; 3 Lien Centre for Palliative Care, Duke-NUS Medical School, Singapore, Singapore; 4 Division of Supportive and Palliative Care, National Cancer Centre, Singapore, Singapore; 5 Department of General Medicine, Sengkang General Hospital, Singapore, Singapore; 6 Department of Social Work, Faculty of Arts and Social Sciences, National University of Singapore, Singapore, Singapore; 7 Department of Geriatric Medicine, Institute of Geriatrics and Active Aging, Tan Tock Seng Hospital, Singapore, Singapore; 8 Department of Palliative Medicine, Tan Tock Seng Hospital, Singapore, Singapore; 9 Center for Child Health Research, Tampere University, Tampere, Finland; 10 Program in Health Services & Systems Research, Duke-NUS Medical School, Singapore, Singapore

**Keywords:** Caregiver, Dementia, Measurement scale, Quality of life, Short form, Singapore caregiver quality of life scale – dementia

## Abstract

**Purpose:**

The Singapore Caregiver Quality of Life Scale – Dementia (SCQOLS-D), developed based on the Singapore Caregiver Quality of Life Scale (SCQOLS), comprises 5 domains and 63 items. It has been shown to be a valid and reliable measurement scale. This study aimed to develop and evaluate a short form of the SCQOLS-D.

**Methods:**

Data were collected from 102 family caregivers of person with dementia in Singapore. Candidate items were shortlisted by factor analysis, correlation and best subset regression. Shortlisted items with the best measurement properties including correlations with the long form and other established measures, internal consistency and test-retest reliability were identified. Their properties were compared with the corresponding domain scores in the long form of SCQOLS-D and a short form of the SCQOLS. A total score based on the average of the domain scores was also evaluated.

**Results:**

A total of fifteen items, two to four items per domain, were selected. The total and domain scores generated from these items strongly correlated with the corresponding score of the long form (correlation coefficients: 0.83–0.97). The short and long forms had comparable correlation coefficients with Negative Personal Impact and Positive Personal Impact measures. The short form showed good internal consistency (Cronbach’s alpha: 0.84–0.92) and test-retest reliability (intra-class correlation coefficient: 0.72–0.93). These 15 items form the SCQOLS-D-15, an abbreviated version of the SCQOLS-D.

**Conclusion:**

The SCQOLS-D-15 showed acceptable measurement properties. This serves as an alternative to the SCQOLS-D to provide rapid assessment of the overall and domain-specific quality of life of caregivers of persons with dementia.

## Introduction

Dementia affects the quality of life (QoL) of not only the patients but also their family caregivers (caregivers in short). Studies revealed that dementia, compared with other chronic diseases like cancer, has both different and common impacts on caregivers. While caregivers of person with dementia (PWD) may have feelings of guilt or shame due to the PWD’s embarrassing behavior arises from the condition [1] and suffered more stress than caregivers of cancer patients [2], they shared similar level of burden and experiences of caregiving process [3–5]. Therefore, there is a demand of dementia-specific instruments to evaluate the QoL of caregivers of PWD. These instruments were mainly developed in western countries, including the Alzheimer’s Carer’s Quality of Life Inventory (ACQLI) in the UK [6], the caregiver QoL scale in the PIXEL dementia study (PIXEL) in France [7] and the Caregiver-targeted Quality of Life Measure (CGQOL) in the USA [8]. They may not be adequate for Asian caregivers due to socio-cultural differences [9]. For example, Asian people are expected to care for their family members who have chronic illness or frailty as much as possible [10]. Research in the USA found that Asian American caregivers spent more time on caregiving than caregivers of other ethnicity [11]. Therefore, the Singapore Caregiver Quality of Life Scale – Dementia (SCQOLS-D) were recently developed for Asian caregivers of PWD [12]. This is an extension of the Singapore Caregiver Quality of Life Scale (SCQOLS) [13, 14] that was developed using data from caregivers of cancer patients by adding 12 dementia-specific items derived from the three abovementioned dementia-specific instruments [6–8] or solicited from semi-structured interviews with caregivers of PWD [12].

To facilitate rapid assessment of caregiver QoL for identification of needy caregivers, short forms have been developed for the 51-item SCQOLS [15]. The 15-item SCQOLS-15, similar to its parent long form [14], provides total and domain scores that evaluate, respectively, the overall and domain-specific QoL for caregivers of cancer patients. The 10-item SCQOLS-10 only assesses overall QoL. Measurement properties of these short forms have been demonstrated [15]. The 63-item SCQOLS-D inherited the 5-domain structure and the scoring algorithm from the SCQOLS [12–14]. It is intuitive to consider the SCQOLS-15 as an abbreviated version of the SCQOLS-D for overall and domain-specific QoL assessment because of the commonality of caregiving experience to these caregivers. However, the impacts of the two diseases on caregiver QoL may be different in some aspects, especially in the domains to which new items were added. Hence, there is a need to consider a new set of items to serve as the short form of the SCQOLS-D.

Therefore, this study was conducted to develop a short form of the SCQOLS-D and evaluate its measurement properties. In particular, it would be desirable if this short form can serve as a quick alternative to assess both overall and domain specific QoL of caregivers of PWD in a way that can facilitate comparison or pooling of findings from studies that use different forms of SCQOLS-D. Therefore, we aimed to retain the 5-domain structure of the SCQOLS-D such that five domain scores and a total score can be derived. Moreover, we also evaluated if the corresponding items of the SCQOLS-15 could be used as the short form of the SCQOLS-D.

## Methods

### Design and setting

This is a secondary analysis of the data collected from 102 family caregivers of PWD for the development and validation of the SCQOLS-D [12]. These caregivers were recruited from January to November 2019 from the Department of Geriatric Medicine of the Singapore General Hospital. Each participant signed an informed consent. This study was approved by the Singapore Health Services Centralized Institutional Review Board (#2018/2896).

The study design has been described in detail previously [12]. In brief, family caregivers of a PWD who were aged 21 years or older and were living with the PWD or spent at least 10 h a week in giving care to the PWD were eligible to participate in this study. We defined a family caregiver as a family member who takes direct care of the PWD’s daily and healthcare needs, ensures the supply of care to meet these needs, or makes decisions on how these needs are met. Each consented caregiver was asked to self-administer a baseline survey, followed by a follow-up survey through prepaid return mail one week after the baseline. Four caregivers who requested interviewer-administration at baseline were not included in this study.

### Questionnaire and measurements

The baseline survey consisted of the SCQOLS-D, questions on caregiver demographics, the Brief Assessment Scale for Caregivers (BASC), and the PWD’s functional staging assessment. The SCQOLS-D has 63 items divided into 5 domains: Physical Well-being (PW; 12 items), Mental Well-being (MW; 18 items), Experience & Meaning (EM; 16 items), Impact on Daily Life (DL; 13 items) and Financial Well-being (FW; 4 items) [12]. Each item is rated on a 5-point scale, from not at all (0) to very much (4). For each domain, we calculate the domain score by first recoding negatively worded items so that a higher score represents a better QoL. Then we take the mean score of the items in that domain and rescale it to a 0–100 scale by multiplying it by 25. Missing values were imputed by the half-rule, i.e. be replaced the mean of other items in the same domain if half or more of the items in that domain were answered [16]. We also obtain a total score by taking a weighted average of the five domain scores, using the number of items in the five domains as the weights. The SCQOLS-D resembles the SCQOLS with two exceptions: (i) the MW and EM domains of SCQOLS-D have, respectively, 8 and 4 additional dementia-specific items, and (ii) SCQOLS-D uses dementia-specific phrases like “my relative with dementia” and “my relative has dementia” to replace generic phrases in SCQOLS on “my sick family member” and “my family member is sick” [12–14].

The baseline questionnaire package also included the BASC, from which a Negative Personal Impact (NPI) and a Positive Personal Impact (PPI) score can be derived [17]. The caregivers were asked to indicate their relationship with the PWD and time spent on taking care of the PWD per week. The PWD’s functional status was evaluated by the Functional Assessment Staging Test (FAST), administered by a researcher assistant [18].

The follow-up survey contained the SCQOLS-D and questions on self-rated change in the caregivers’ QoL and whether the PWD had visited an emergency department or been admitted to/discharged from a hospital/medical facility since the baseline interview.

### Development and evaluation of short form

**Fig. 1:**
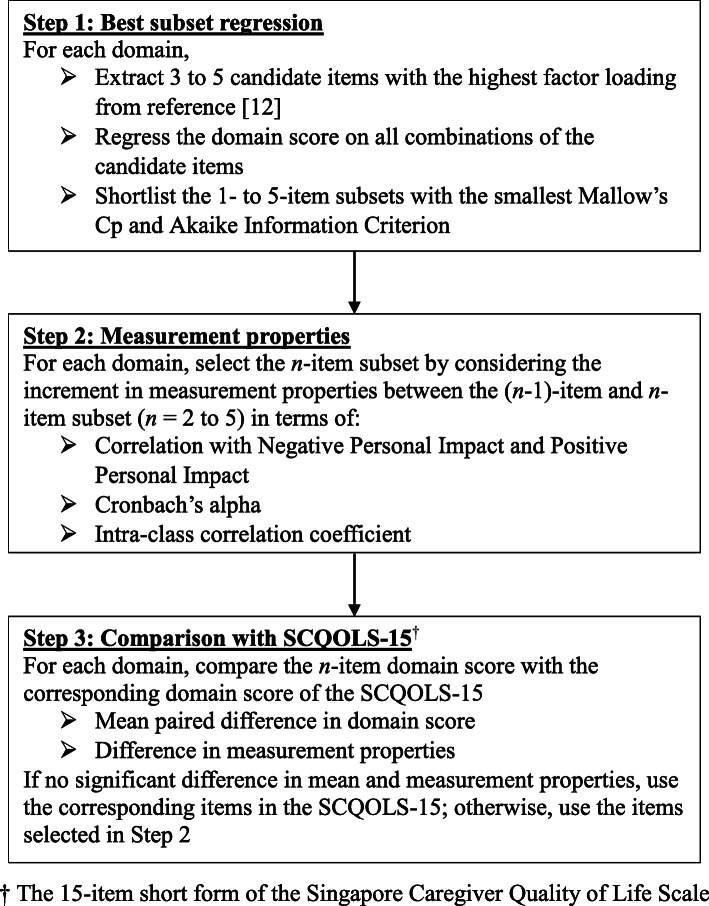
Process of developing the short form of the Singapore Caregiver Quality of Life Scale – Dementia

The development of the short form of the SCQOLS-D included three steps (Fig. 1). Firstly, we identified items with the highest factor loadings of each domain from the previously published factor analysis on the long form as candidate items [12, 19, 20]. The factor analysis was performed by Mplus using the Weighted Least Squares method for data with Missing Values (WLSMV) [21]. Based on the previous literature, we expected that, the number of items for each domain needed to sufficiently capture most of the information would be two to five and proportional to the number of items in that domain in the long form [22, 23]. Therefore, we identified five items from MW and EM, four items from PW and DL and three items from FW as candidate items. Then, for each of 1-, 2-, 3-, 4- (except FW) and 5-item (MW and EM) subsets, we computed the mean of the item scores and examined its Pearson correlation coefficient (r) with the corresponding domain score using the baseline data, and also regressed the domain score of the long form on all subsets of the candidate items through linear regression [19, 20]. Since the responses are of ordinal type in nature, we also computed the Spearman’s and polychoric correlation coefficients as a supplementary analysis. Two sets of linear regression analyses were performed, respectively treating the candidate items as continuous and categorical variables. Except for 1-item subsets, any item with a missing value was imputed by the half-rule prior to the regression and correlation analyses. The best subsets with 1, 2, 3, 4 and 5 items, identified by the smallest Mallow’s Cp and Akaike Information Criterion (AIC) among the models with the same number of items, were shortlisted to enter the next step.

The second step examined the measurement properties of the domain score derived from the items of the shortlisted best subsets. For each domain and each number of items *n* (from 1 to 5), an *n*-item domain score was calculated by the mean of the item scores multiplied by 25. The correlation of the domain score with the NPI and PPI, and the internal consistency of the items in each of the shortlisted 2- to 5-item subsets were estimated using the baseline data. Since the responses to the items are of ordinal data type, we used the ordinal version of the Cronbach’s alpha in the evaluation of internal consistency [24]. We also assessed the test-retest reliability of the domain scores by estimating the intra-class correlation coefficient (ICC) using both baseline and follow-up data. To ensure the caregiver condition was stable at the two time points, data were included in the estimation of ICC only if the caregiver had returned the follow-up survey within 21 days from baseline, reported no self-rated change in QoL and the PWD had neither visited an emergency department nor been admitted to/discharged from a hospital/medical facility since baseline. A subset would be considered not acceptable if its correlations with the NPI and PPI substantially deviated from those of the long form. Since there is no universally agreed threshold of Cronbach’s alpha and ICC for an acceptable level of internal consistency and test-retest reliability, respectively, we chose the subset by considering the increment of these measures from the (*n*–1)-item subset to the *n-*item subset. After comparing the measurement properties, the number of items *n* to be included in the short form was determined for each domain.

The selected *n*-item domain score was then compared with the corresponding domain score of the SCQOLS-15 in the third step. This is to evaluate whether the dementia-specific selection of items to generate a short form is essential, or using the items corresponding to the SCQOLS-15 that were developed from a sample of caregivers of cancer patients is sufficient. A 95% confidence interval (CI) for matched samples was constructed for the mean paired difference between the two domain scores, whereas a 95% bootstrap CI for the difference in each of the above-mentioned measurement properties was estimated based on 1000 replicates. For each domain, if there was no significant difference in the mean score and all measurement properties, the items corresponding to the SCQOLS-15 would be chosen as the short form items of the SCQOLS-D for that domain; otherwise, the short form would comprise the *n* items selected in the second step.

After determining the items to be included in the short form for each domain, a total score was generated by the weighted average of the five domain scores, using the number of items in the five domains in the long form as the weights. These weights retain the relative importance of each domain to the total score, ensuring its compatibility between the long and short forms.

To investigate whether comparable measurement properties could be reproduced by another set of data, the correlation between the domain/total scores of the long and short forms and the Cronbach’s alpha of the short form items were estimated using the follow-up data and evaluated against those obtained from the baseline data.

## Results

### Participant characteristics

The characteristics of the 102 caregivers have been summarized previously [12]. In brief, the sample mean age was 54.6 (standard deviation 10.6, range 22–86) years; 80.4% were female; 88.2% were ethnic Chinese; 70.6% received tertiary education or above; 86.3% were the son/daughter of the PWD. They spent an average of 43.5 h on caregiving duties per week. Half of the PWD had mild functional limitations indicated by a FAST score of 5 or below.

### Correlation of domain scores between long and short forms

Table 1 presents the candidate items of each domain with the corresponding factor loading. The regression model specifying the candidate items as categorical variables with the smallest Mallow’s Cp and AIC among the subsets with same number of items for each domain was identified as the best subset and is shown in Table 2. Results from models with the candidate items being continuous variables were identical in terms of the rankings of the Cp and AIC (details not shown). Unlike the other items, DL8 (“*Affected career development*”) has an option of “Not Applicable”. The SCQOLS-D scoring algorithm considers this a missing value. Twenty-two (21.6%) caregivers thus had a missing value in this item. For the 2-, 3- and 4-item subsets of DL, the missing value in DL8 was imputed by the half-rule; but for the 1-item subsets, these caregivers were excluded from the analysis. Table 2 also shows the correlation between the mean score of the subsets of candidate items and the long form domain score. Except the 1-item subset of MW, all shortlisted subsets had a correlation coefficient larger than 0.8. The supplementary analysis showed that, compared to the Pearson’s correlation coefficients, the Spearman’s correlation coefficients were consistently smaller by 0.02 to 0.06 and the polychoric correlation coefficients were larger by 0.02 to 0.08 without altering the ranking of the observed correlation coefficients, hence led to the same shortlisted subsets.

**Table 1:** Candidate items for short form identified from exploratory factor analysis of the Singapore Caregiver Quality of Life Scale – Dementia (SCQOLS-D)

Domain	Item number	Brief description	Factor loading^a^
Physical Well-being	PW6	Poor appetite	0.85
	PW7	Weight loss	0.84
	PW8	Weakened body	0.91
	PW11	Difficulty remembering things	0.85
Mental Well-being	MW4	Hopelessness	0.86
	MW5	Helplessness	0.85
	MW7	Feel angry	0.85
	MW8	Feel frustrated	0.92
	MW18	My relative with dementia is a burden	0.84
Experience & Meaning	EM4	Thankful for the good things	0.82
	EM5	Willing to make the best	0.81
	EM6	Satisfaction from caring	0.83
	EM7	Experience of positive changes	0.79
	EM12	Family appreciation	0.86
Impact on Daily Life	DL3	Not satisfied with time to myself	0.96
	DL4	No time for recreational activities	0.92
	DL5	Not able to do what I want	0.95
	DL8	Affected career development	0.94
Financial Well-being	FW1	Depleting savings	0.96
	FW3	Uncertain about future financial situation	0.92
	FW4	Personal spending restricted	0.91

^a^Extracted from Online Supplementary Material S1 of reference [12]

**Table 2:** Mallow’s Cp, Akaike Information Criterion (AIC) and Pearson’s correlation coefficient (r) with the long form domain score of the selected *n*-item subsets for each domain

Domain	Items	Cp	AIC	r
Physical Well-being	PW8	115.7	745.2	0.85
	PW8 + PW11	51.7	709.3	0.90
	PW6 + PW8 + PW11	17.5	680.8	0.93
	PW6 + PW7 + PW8 + PW11	15.0	676.5	0.93
Mental Well-being	MW8	72.0	773.1	0.75
	MW5 + MW8	26.7	748.1	0.83
	MW5 + MW8 + MW18	17.2	738.6	0.83
	MW4 + MW5 + MW8 + MW18	17.7	738.0	0.83
	MW4 + MW5 + MW7 + MW8 + MW18	22.0	741.0	0.83
Experience & Meaning	EM6	105.4	774.9	0.80
	EM6 + EM12	36.8	739.9	0.88
	EM5 + EM6 + EM12	15.0	718.9	0.91
	EM5 + EM6 + EM7 + EM12	16.0	718.3	0.91
	EM4 + EM5 + EM6 + EM7 + EM12	22.0	723.8	0.91
Impact on Daily Life	DL5	116.7	596.1	0.90
	DL5 + DL8	81.7	727.7	0.93
	DL4 + DL5 + DL8	19.8	679.2	0.95
	DL3 + DL4 + DL5 + DL8	23.0	680.2	0.95
Financial Well-being	FW3	344.2	780.3	0.92
	FW1 + FW3	51.0	675.6	0.97
	FW1 + FW3 + FW4	13.0	641.1	0.98

### Measurement properties of candidate short forms

Table 3 shows, for each shortlisted subset and all items in the long form, the correlation of the mean item score with the NPI (r(NPI)) and PPI (r(PPI)), Cronbach’s alpha and ICC. Among the 102 caregivers, 50 completed the follow-up survey, of whom 34 satisfied the inclusion criteria for the assessment of test-retest reliability and thus included in the estimation of ICC.

**Table 3:** Measurement properties of the long form and selected *n*-item subsets for each domain

Domain	Items	r(NPI)	r(PPI)	Alpha	ICC
Physical Well-being	All 12 items (long form)	0.60	0.12	0.92	0.86
	PW8	0.55	−0.09		0.65
	PW8 + PW11	0.55	0.06	0.75	0.73
	PW6 + PW8 + PW11	0.58	0.06	0.84	0.81
	PW6 + PW7 + PW8 + PW11	0.59	0.07	0.90	0.82
Mental Well-being	All 18 items (long form)	0.64	0.11	0.91	0.89
	MW8	0.57	0.09		0.80
	MW5 + MW8	0.67	0.17	0.80	0.90
	MW5 + MW8 + MW18	0.70	0.22	0.82	0.93
	MW4 + MW5 + MW8 + MW18	0.68	0.26	0.88	0.92
	MW4 + MW5 + MW7 + MW8 + MW18	0.67	0.25	0.91	0.90
Experience & Meaning	All 16 items (long form)	0.28	0.44	0.92	0.75
	EM6	0.21	0.50		0.71
	EM6 + EM12	0.30	0.47	0.68	0.76
	EM5 + EM6 + EM12	0.31	0.44	0.78	0.72
	EM5 + EM6 + EM7 + EM12	0.31	0.48	0.85	0.72
	EM4 + EM5 + EM6 + EM7 + EM12	0.31	0.44	0.88	0.74
Impact on Daily Life	All 13 items (long form)	0.76	0.06	0.96	0.87
	DL5	0.65	0.13		0.77
	DL5 + DL8	0.69	0.09	0.85	0.80
	DL4 + DL5 + DL8	0.70	0.12	0.90	0.78
	DL3 + DL4 + DL5 + DL8	0.70	0.11	0.94	0.77
Financial Well-being	All 4 items (long form)	0.58	−0.08	0.95	0.86
	FW3	0.54	− 0.12		0.77
	FW1 + FW3	0.61	−0.05	0.89	0.79
	FW1 + FW3 + FW4	0.60	−0.08	0.94	0.83

Abbreviations: *r(NPI) and r(PPI)* Pearson’s correlation coefficients with Negative Personal Impact and with Positive Personal Impact; *ICC* intraclass correlation coefficient

For PW, the 3-item subset had r(NPI) and r(PPI) comparable to those of the long form, and showed a large increment compared with the 2-item subset in the Cronbach’s alpha (0.84 vs 0.75) and ICC (0.81 vs 0.73). Further increasing the number of items to four did not substantially improve the measurement properties. Hence, the 3-item subset was selected for PW.

For MW, the 3-item subset was comparable with the 4-item subset in r(NPI) (0.70 vs 0.68) and test-retest reliability (ICC: 0.93 vs 0.92), albeit less well in Cronbach’s alpha (0.88 vs 0.82). However, the 3-item subset obtained a r(PPI) (0.22) closer to that of the long form (0.11) than the 4-item subset (0.26). Taking parsimony into consideration, we selected the 3-item subset for MW.

Both the 3- and 4-item subsets of EM had similar r(NPI), r(PPI) and ICC, but the 4-item subset had a remarkably better Cronbach’s alpha (0.85) than the 3-item subset (0.78). Therefore, the 4-item subset was selected for EM.

Since DL8 was involved in the 2-, 3- and 4-item subsets of the DL domain, the Cronbach’s alpha for these subsets was estimated based on the 80 caregivers who had no missing values in DL8. While r(NPI), r(PPI) and ICC of the 2- and 3-item subsets were similar, the 3-item subset markedly outperformed the 2-item subset in Cronbach’s alpha (0.90 vs 0.85). Hence, the 3-item subset was selected.

For FW, the 2- and 3-item subsets had similar r(NPI) and r(PPI) to those of the long form. Since the 2-item subset obtained a sufficiently high Cronbach’s alpha (0.89) and ICC (0.79), it was selected due to parsimony despite the increment in these measures in the 3-item subset.

### Comparison with SCQOLS-15 domain scores

The items selected for the short form of the SCQOLS-D were the same as those in the SCQOLS-15 in FW but different in the other four domains. The difference in mean scores generated by the selected items minus that generated by the SCQOLS-15 items for these domains were compared (Table 4). Difference in mean score was significant in PW, MW and EM, but insignificant in DL (− 0.65, 95% CI = − 2.37 to 1.06). Significant differences in measurement properties estimated by bootstrap resamples were found in MW and EM, but not in PW and DL (Table 4). The MW score of the selected items had r(NPI) (0.70) and r(PPI) (0.22) more similar to those of the long form (0.64 and 0.11) than those of the items in the SCQOLS-15 (0.54 and − 0.09), and a significantly higher ICC (difference: 0.18, 95% bootstrap CI = 0.10 to 0.26). For EM, r(PPI) of the selected items (0.48) was also closer to the long form’s r(PPI) (0.44) than that of the SCQOLS-15 EM score (0.39). Therefore, the short form will use the selected items for PW (3 items), MW (3 items) and EM (4 items); and for DL, the three items (DL2 + DL4 + DL5) in the SCQOLS-15 will be used. Together with the two items in FW, there are 15 items in the short form for the SCQOLS-D, or SCQOLS-D-15 in short.

**Table 4:** Comparison of mean score and measurement properties between the selected items and the items in the 15-item short form of the Singapore Caregiver Quality of Life Scale (SCQOLS-15) developed in a previous study of caregivers of cancer patients

	Mean score	r(NPI)	r(PPI)	Alpha	ICC
Domain and items	Estimate (95% CI^a^)	Estimate (95% CI^b^)	Estimate (95% CI^b^)	Estimate (95% CI^b^)	Estimate (95% CI^b^)
Physical Well-being					
Selected items PW6 + PW8 + PW11	80.3	0.58	0.06	0.84	0.81
SCQOLS-15 PW4 + PW6 + PW8	77.5	0.58	0.04	0.89	0.79
Difference	2.86 (1.13, 4.59)	0.00 (−0.07, 0.07)	0.02 (−0.06, 0.11)	− 0.04 (− 0.11, 0.02)	0.01 (− 0.03, 0.06)
Mental Well-being					
Selected items MW5 + MW8 + MW18	74.5	0.70	0.22	0.82	0.93
SCQOLS-15 MW2 + MW3 + MW8	55.0	0.54	− 0.09	0.67	0.76
Difference	19.5 (15.9, 23.1)	0.16 (0.04, 0.29)	0.31 (0.12, 0.50)	0.14 (−0.01, 0.28)	0.18 (0.10, 0.26)
Experience & Meaning					
Selected items EM5 + EM6 + EM7 + EM12	61.6	0.31	0.48	0.85	0.72
SCQOLS-15 EM4 + EM7 + EM11 + EM12	59.6	0.36	0.39	0.80	0.74
Difference	2.04 (0.24, 3.85)	−0.05 (−0.13, 0.03)	0.09 (0.01, 0.17)	0.05 (0.001, 0.10)	−0.02 (− 0.08, 0.04)
Impact on Daily Life					
Selected items DL4 + DL5 + DL8	69.9	0.70	0.12	0.90	0.78
SCQOLS-15 DL2 + DL4 + DL5	70.6	0.69	0.15	0.92	0.78
Difference	−0.65 (−2.37, 1.06)	0.02 (− 0.04, 0.07)	−0.03 (− 0.10, 0.04)	−0.01 (− 0.06, 0.04)	0.01 (− 0.04, 0.06)

Abbreviations: *r(NPI) and r(PPI)* Pearson’s correlation coefficients with Negative Personal Impact and with Positive Personal Impact; *ICC* intraclass correlation coefficient; *CI* confidence interval

^a^Confidence interval for paired difference in matched samples

^b^Bootstrap confidence interval for difference based on 1000 replicates

After establishing the SCQOLS-D-15, a total score was computed by taking a weighted average of the five domain scores. The total score of the SCQOLS-D-15 obtained a correlation of 0.89 with that of the long form, a r(NPI) of 0.78 (long form: 0.79), a r(PPI) of 0.29 (0.23), a Cronbach’s alpha of 0.91 (0.96) and an ICC of 0.89 (0.91).

### Reproducibility of measurement properties

The correlation of the SCQOLS-D-15 domain/total scores with the corresponding long form score and the Cronbach’s alpha were reproduced using data from the follow-up survey (Table 5). For the domain scores, the correlation ranged from 0.76 (MW) to 0.97 (FW), which were similar to those from the baseline data with a maximum (absolute) difference of 0.07 in MW (0.83). The Cronbach’s alpha using the follow-up data ranged from 0.83 (MW) to 0.91 (FW), with a maximum difference of 0.06 in PW (0.90 vs 0.84) from the baseline data. The total score had a correlation of 0.94 (baseline: 0.95) with the long form and a Cronbach’s alpha of 0.91 (0.91).

**Table 5:** Measurement properties of the 15-item short form of the Singapore Caregiver Quality of Life Scale – Dementia (SCQOLS-D-15) in the follow-up survey

Domain / Total score	SCQLS-D-15 items	r	Alpha
Physical Well-being	PW6 + PW8 + PW11	0.93	0.90
Mental Well-being	MW5 + MW8 + MW18	0.76	0.83
Experience & Meaning	EM5 + EM6 + EM7 + EM12	0.91	0.85
Impact on Daily Life	DL2 + DL4 + DL5	0.92	0.88
Financial Well-being	FW1 + FW3	0.97	0.91
Total score	15 items above	0.94	0.91

Abbreviation: *r* Pearson’s correlation coefficient of the short form domain / total score with the corresponding score in the long form

## Discussion

We have developed an abbreviated version of the SCQOLS-D for rapid evaluation of QoL of Asian family caregivers of PWD. The development of long, original version of QoL measurement scales usually reflects the intention to achieve a high level of content validity. But it is at the expense of user-friendliness. Similar to previous studies on the development of QoL short forms [15, 22, 23], we found that keeping two to four items per domain was sufficient to keep most of the information and produce assessment results comparable to those from using the parent version. From the viewpoint of developing short measures for quick assessment, the availability of a longer parent version is useful in providing the benchmark to evaluate how short a short form can be without significant loss.

The domain and total scores of the SCQOLS-D-15 were strongly correlated with the long form (Pearson’s correlation coefficient: 0.83–0.97). The scores also demonstrated comparable concurrent validity with the long form (comparable correlations with NPI and PPI) and moderate to substantial internal consistency (Cronbach’s alpha: 0.76–0.90) and test-retest reliability (ICC: 0.72–0.93).

In this study, we selected the subset of items from each domain of the SCQOLS-D by best subset regression and examining their measurement properties in this sample, then compared between this selected subset and the corresponding domain of the SCQOLS-15. For DL, no significant difference in the mean domain score and measurement properties was found between these two subsets. The final set we recommend to form a short version of SCQOLS-D include two items (DL4 and DL5) that are common in both subsets, while the third item we recommend is DL2 (“*Unable to leave home or hospital*”) in the SCQOLS-15 instead of DL8 (“*Affected career development*”) that was shortlisted based on factor analysis and best subset regression in this sample [17]. Given the high rank in factor loading (Table 1), one may expect the subsets including DL8 should have had better measurement properties. A possible reason of the insignificant differences between this subset and the subset developed to form the SCQOLS-15 may be due to the way missing values was handled. The factor analysis used the WLSMV which fully utilizes the data [18]. In this sample, 86.3% of the caregivers were the sons/daughters of the PWD, many of whom were working adults, as compared to 46.2% in the previous study of caregivers of cancer patients, from which the SCQOLS-15 was developed [15]. This demographic profile tended to highlight the importance of DL8. In contrast, the practice of QoL assessment needs the half-rule to compute the domain score. Work-related information carried by DL8 could not be retrieved by DL4 and DL5, thus affected the measurement properties. We estimated the measurement properties for DL using the 80 caregivers who had no missing values in DL8 (details not shown). The shortlisted subset outperformed the subset from the SCQOLS-15 in the correlations with NPI and PPI in this complete-case analysis. This suggests that DL8 may be a better choice than DL2 if working young adults are the target users. However, this study aimed to develop a short form for all caregivers regardless of their age and employment status. The half-rule is a practical approach to deal with missing values, being used or recommended to use in many QoL instruments [16]. We do not notice any user-friendly alternative. Therefore, for the short form of SCQOLS-D, we recommend DL2 which does not involve a “Not Applicable” response and reduces the chance of being unable to calculate the domain score due to missing values beyond imputation by the half-rule.

The development of SCQOLS-D was made by adding 12 items to the SCQOLS. These new items were derived from our pilot study as well as three dementia-specific QoL scales. The ACQLI and PIXAL may not accurately reflect caregivers’ well-being since they focus on negative caregiving experience [6, 7], while the CGQOL does not assess caregivers’ physical well-being [8]. Moreover, there is no financial-related domain in these scales; only one item in the PIXAL asks about caregivers’ whether the respondent’s financial situations are harder to manage since taking care of the relative with PWD, which is embedded in the domain of relation to environment [7]. Compared with these scales, the SCQOLS-D-15, which inherits the domain structure of the SCQOLS-D, provides a more comprehensive content coverage.

The SCQOLS-D-15 can be completed quickly and thus reduces respondent burden and enhances the efficiency in evaluating the QoL of caregivers of PWD. This is especially appropriate in clinical settings or studies that involve many instruments, and longitudinal studies with multiple time points, to avoid imposing additional burden on the respondents. As a consequence of item reduction, there may be some degrees of loss in content validity in short forms when compared to their parent long forms. The SCQOLS-D-15 is of no exception. Investigators have to understand the pros and cons of both long and short forms and choose the suitable one according to their needs and study design. However, the 5-domain structure fitted the SCQOLS-D data well; the items within the same domain do share similarity and relatedness. Careful selection of some representative items does not appear to compromise the content validity much. For example, although hopelessness and helplessness are different constructs and the latter but not the former is kept in the MW domain of SCQOLS-D-15, the items in the short form MW domain appear to have a good representation of the overall profile of items in the long form MW domain. A relatively visible difference is that, as discussed above in more details, the career related items in the DL domain of SCQOLS-D are not included in the SCQOLS-D-15. Studies that focus on younger caregivers and career issues may want to include additional measures to complement SCQOLS-D-15.

A limitation of the present study is that we collected the data using the long form and extracted items from it to develop the short form. The interpretation here needs to assume that the selected items have the same performance no matter if they are embedded in the long form SCQOLS-D as in this study or administered as a standalone short form. Previous studies have illustrated that QoL assessment was usually free of such a context effect [25–28]. Hence, we take this assumption as valid. Another limitation is the relatively small proportion of non-Chinese caregivers. Moreover, some aspects of measurement properties such as responsiveness to change have not been evaluated. Further studies aiming to assess the measurement properties comprehensively and using the SCQOLS-D-15 as a standalone instrument and with broader ethnicity representation are warranted.

In conclusion, a 15-item short form of the SCQOLS-D was developed. The short form is an alternative to the SCQOLS-D with acceptable measurement properties that provides quick assessment of overall and domain-specific QoL of caregivers to PWD. This can facilitate clinical practice, program evaluation and research on caregivers of PWD.

## Data Availability

The dataset is not publicly available due to IRB restrictions but are available from the last author on reasonable request.
